# Recapitulated Crosstalk between Cerebral Metastatic Lung Cancer Cells and Brain Perivascular Tumor Microenvironment in a Microfluidic Co‐Culture Chip

**DOI:** 10.1002/advs.202201785

**Published:** 2022-06-03

**Authors:** Hyunho Kim, Jason K. Sa, Jaehoon Kim, Hee Jin Cho, Hyun Jeong Oh, Dong‐Hee Choi, Seok‐Hyeon Kang, Da Eun Jeong, Do‐Hyun Nam, Hakho Lee, Hye Won Lee, Seok Chung

**Affiliations:** ^1^ School of Mechanical Engineering, College of Engineering Korea University Seoul 02841 Republic of Korea; ^2^ Center for Systems Biology Massachusetts General Hospital Boston MA 02114 USA; ^3^ Department of Biomedical Sciences Korea University College of Medicine Seoul 02841 Republic of Korea; ^4^ George W. Woodruff School of Mechanical Engineering Georgia Institute of Technology Atlanta GA 30332 USA; ^5^ Department of Biomedical Convergence Science and Technology Kyungpook National University Daegu 41566 Republic of Korea; ^6^ Cell and Matrix Research Institute Kyungpook National University Daegu 41944 Republic of Korea; ^7^ Bioscience division, Life Sciences and Laboratory Products Group Thermo Fisher Scientific Solutions Seoul 06349 Republic of Korea; ^8^ Institute for Refractory Cancer Research Samsung Medical Center Seoul 06351 Republic of Korea; ^9^ Department of Health Science & Technology, Samsung Advanced Institute for Health Sciences & Technology (SAIHST) Sungkyunkwan University Seoul 06351 Republic of Korea; ^10^ Department of Neurosurgery, Samsung Medical Center Sungkyunkwan University School of Medicine Seoul 06351 Republic of Korea; ^11^ Department of Urology, Center for Urologic Cancer National Cancer Center Goyang 10408 Republic of Korea; ^12^ KU‐KIST Graduate School of Converging Science and Technology Korea University Seoul 02841 Republic of Korea

**Keywords:** brain perivascular tumor microenvironment, cerebral metastatic lung cancer cells, microfluidic co‐culture chip

## Abstract

Non‐small cell lung carcinoma (NSCLC), which affects the brain, is fatal and resistant to anti‐cancer therapies. Despite innate, distinct characteristics of the brain from other organs, the underlying delicate crosstalk between brain metastatic NSCLC (BM‐NSCLC) cells and brain tumor microenvironment (bTME) associated with tumor evolution remains elusive. Here, a novel 3D microfluidic tri‐culture platform is proposed for recapitulating positive feedback from BM‐NSCLC and astrocytes and brain‐specific endothelial cells, two major players in bTME. Advanced imaging and quantitative functional assessment of the 3D tri‐culture model enable real‐time live imaging of cell viability and separate analyses of genomic/molecular/secretome from each subset. Susceptibility of multiple patient‐derived BM‐NSCLCs to representative targeted agents is altered and secretion of serpin E1, interleukin‐8, and secreted phosphoprotein 1, which are associated with tumor aggressiveness and poor clinical outcome, is increased in tri‐culture. Notably, multiple signaling pathways involved in inflammatory responses, nuclear factor kappa‐light‐chain‐enhancer of activated B cells, and cancer metastasis are activated in BM‐NSCLC through interaction with two bTME cell types. This novel platform offers a tool to elucidate potential molecular targets and for effective anti‐cancer therapy targeting the crosstalk between metastatic cancer cells and adjacent components of bTME.

## Graphical abstract

The development of a microfluidic triculture chip to simulate interactions between brain metastatic tumor cells and the microenvironment of the brain tumor. The microfluidic chip allows for the cultivation of patient‐derived cells in complex components of reconstituted brain, as well as the spatial collection of conditioned media and cells for a variety of biological analyses. This innovative platform allows the screening of drugs that target the intercellular communication between metastatic cancer cells and microenvironmental components at the metastatic site.

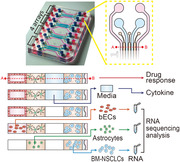

## 1 Introduction

In spite of improved diagnostic techniques and advanced therapeutic strategies for the increased incidence of brain metastasis (BM),^[^ ^1^, ^2^, ^3^ ^]^ BM in patients with non‐small cell lung carcinoma (NSCLC) is life‐threatening and associated with poor survival and high morbidity with limited therapeutic options.^[^ ^4^, ^5^, ^6^ ^]^ A substantial portion of NSCLCs has been found to harbor specific driver alterations affecting tumor progression, making them sensitive to the inhibition of the corresponding oncogenic pathway by targeted therapies.^[^ ^4^, ^7^, ^8^ ^]^ For example, advent of targeted systemic agents for the mutated epidermal growth factor receptor (EGFR) or anaplastic lymphoma kinase (ALK) rearranged NSCLC has renewed interest in utilizing system therapy.^[^ ^9^ ^]^ These mutations activate essential signaling pathways, including cyclin‐dependent kinase (CDK), phosphoinositide 3‐kinase‐serine/threonine‐specific protein kinase‐mammalian target of rapamycin (PI3K/Akt/mTOR) pathway, Receptor tyrosine‐protein kinase erbB‐2 (ERBB2), EGFR, and mitogen‐activated protein kinase (MAPK) pathway, which predicts sensitivity to targeted therapies.^[^ ^6^ ^]^ However, an increased likelihood of resistance to the drugs could stem from evolved treatment‐refractory metastatic NSCLC tumor cells by the tumor microenvironment (TME).^[^ ^3^, ^5^, ^10^ ^]^ Brain metastatic NSCLC (BM‐NSCLC) tumor cells form dynamic interactions with brain TME in the creation of a brain metastatic niche, known to be responsible for initiating metastasis on the tumor cells’ ability to survive inside and resulting in unexpected response to targeted therapies.^[^ ^6^ ^]^ BM‐NSCLC cells utilize the brain TME to sustain the viability and growth of established metastases.

**Figure 1:**
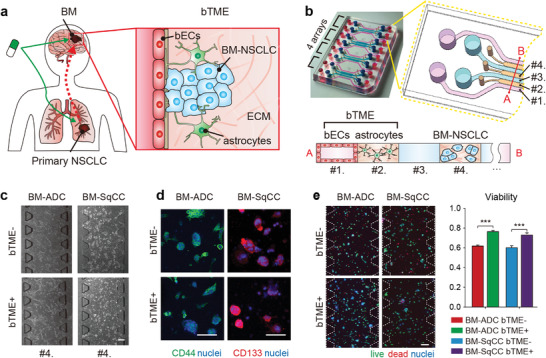
Schematic illustration of brain metastatic niche of NSCLC cells and microfluidic device for recapitulating the niche. a) The brain metastatic niche involving bTME with bECs and astrocytes. b) Representative photography and drawing of the microfluidic device with seven channels, and its cross‐sectional illustration with three types of cells. c) Phase‐contrast images of tumor cells monocultured (bTME‐) and co‐cultured (bTME+) in a microfluidic device. d) Representative fluorescence images of patient tumor cells of cancer stemness markers. e) Representative fluorescence images of patient tumor cells tagged by live/dead kit (left), and measured viability (right). Scale bars indicate 200 µm. The viability data presented as average values ± SEM, n = 65 for BM‐ADC and n = 21 for BM‐SqCC.

The foreign soil in which the escaping tumor cells survive includes preexisting nonmalignant cell types, such as brain endothelial cells (bECs) and astrocytes^[^ ^9^ ^]^ (**Figure** **1**a). These two stromal players in brain metastatic niche may influence BM‐NSCLC cells’ survival, dormancy, and even stemness promotion.^[^ ^5^, ^6^ ^]^ For example, islands of reactive astrocytes forming peritumoral astrogliosis can promote anchorage‐independent growth of tumor cells and tumor growth through paracrine signaling.^[^ ^5^, ^6^, ^11^ ^]^ Endothelin peptides from astrocytes initiated by tumor cell‐driven activation of the MAPK and AKT pathways can also promote endothelin‐driven astrocytic survival by upregulating anti‐apoptotic genes in tumor cells, thereby promoting treatment resistance.^[^ ^5^, ^12^ ^]^ At the same time, the complex interconnections in perivascular TME could contribute to the modification of the gene expression profile of cells in TME, which causes morphological change.^[^ ^13^ ^]^ The complexity makes it difficult to anticipate the response of BM‐NSCLC patients to therapeutic drugs, emphasizing the need to determine the exact nature of the bidirectional interactions and characterize several molecules at the metastatic site.^[^ ^14^ ^]^

Due to the absence of a preclinical platform recapitulating the real brain metastatic niche, we developed an in vitro microfluidic cell culture assay, which can quantify the response to drugs by tumor cells and molecular or morphological traces under cell‐cell and cell‐TME interactions, which conventional 2D assays cannot.^[^ ^15^ ^]^ Impactful capability of the assay exists in the precise orchestration of biochemical reactions in TME on molecular, cellular, and tissue scales in high throughput manner.^[^ ^16^ ^]^ Microfluidic scale offers important features for TME reconstitution via self‐organization of cultured cells and spatiotemporal control of physical and biochemical influencers, combining emerging advances of multiple types of cells with compartmentalized ECM hydrogels.^[^ ^16^ ^]^

However, microfluidic screening of drugs with patient‐derived tumor cells (PDCs) remains at the preliminary stage to reconstitute complicated communications and interactions between tumor cells and components in TME and acquire abundant biological analysis.^[^ ^14^ ^]^ This study proposes a novel strategy to recapitulate the metastatic brain niche by tri‐culturing PDCs of a BM‐NSCLC patient with astrocytes and bECs forming a reconstituted brain tumor microenvironment (bTME). Drug screening and biological analysis including high content imaging and molecular profiling provided precise drug screening for PDCs under local enrichment of paracrine factors secreted from stromal cells in TME considering co‐evolution of BM‐NSCLC cells and tumor‐associated bTME components.

## 2 Results

### 2.1 Microfluidic Tri‐Culture of Patient‐Derived Tumor Cells Under Brain Microenvironment

BM‐ADC (PDCs isolated from BM in a 65‐yr‐old female patient with lung adenocarcinoma (ADC)) and BM‐SqCC (PDCs isolated from BM in a 68‐yr‐old old male patient with lung squamous cell carcinoma (SqCC)) cells were selected to represent two typical BM‐NSCLC cases.^[^ ^7^, ^17^ ^]^ The PDCs were confirmed to preserve genomic similarities with their original tissues.^[^ ^18^ ^]^ Immortalized human cerebral microvascular endothelial cells (hCMEC/D3) and primary human brain astrocyte (ACBRI 371) cells were selected to model bTME. A microfluidic device was designed to culture three types of cells in a spatially organized manner, which has four units, each of which has four medium channels (Figure 1b, channels #1 and #3) divided by three hydrogel channels (channels #2 and #4). BM‐ADC or BM‐SqCC cells were suspended in type 1 collagen hydrogel (COL1) solution and then introduced into the center hydrogel channel #4, gelled and supplemented by neurobasal medium in medium channel #3 (Figure 1b). COL1 mixed with ACBRI 371 cells introduced into another hydrogel channel #2, on which hCMEC/D3 cells were seeded after gelation in medium channel #1. BM‐NSCLC cells were spatially compartmentalized in the microfluidic channels (Figure 1d), monocultured (bTME‐) or co‐cultured with hCMEC/D3 and ACBRI 371 cells (bTME+) (Figure 1c and Figure S1a, Supporting Information) All medium channels were filled by cancer cell culture medium consisted serum‐free neurobasal medium with growth factors (B27, N2, L‐glutamine, epidermal growth factor (EGF), basic fibroblast growth factor (bFGF), recombinant human IGF‐1 (rhIGF‐1), and recombinant human neuregulin‐1 beta1/heregulin‐beta1 (rhNRG‐1‐b1/HRG‐b1) to preserve cancer stem cell characteristics of the BM‐NSCLC cells.

The stemness of BM‐NSCLC cells was confirmed by CD44 and CD133 under both bTME‐ and bTME+ conditions (Figure 1d).^[^ ^18^ ^]^ The medium in each medium channel was refreshed daily for 7 days. CD31 and GFAP were checked in the hCMEC/D3 and ACBRI 371 cells, respectively (Figure S1b, Supporting Information). Previous studies reported promoted viability of tumor cell lines when co‐cultured with ECs.^[^ ^13^, ^19^, ^20^ ^]^ BM‐NSCLC cells used in this work also presented enhanced viability under bTME+ condition for 7 days (Figure 1e). However, number and size of BM‐NSCLC aggregate under both bTME+ and bTME‐ conditions did not present statistical differences (Figure S2, Supporting Information).

**Figure 2:**
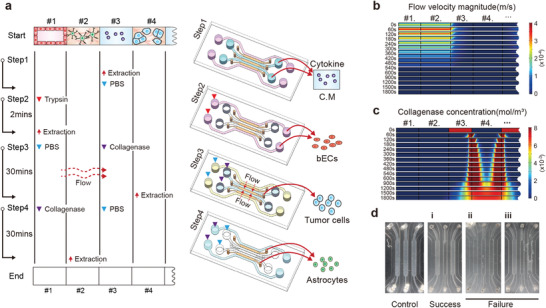
Protocol for isolated extraction of medium and cells from microfluidic device. a) Whole sequences used to collect medium and cells individually. The process of separating cells and medium from the chip consists of a total of 4 steps. Step1 is extracting conditioned media (C.M). PBS washing was required twice between every step. Step 2 is extracting the bECs cultured on the bottom of the channel, and cells were detached using trypsin. Step 3 is extracting tumor cells embedded in collagen; 120 µL of 4 °C PBS was added to channel #1, and 60 µL of 37 °C collagenase was added to channel #3. A hydrostatic pressure of 60 µL forms a flow during incubation. Step 4 is extracting the astrocyte in channel #2; 60 µL of 37 °C collagenase and 60 µL of 37 °C PBS were added to channels #1 and #3, respectively, and incubated for 30 min, following which astrocytes were extracted. The PBS and enzyme solution injection channel at each step are shown in the schematic illustration on the right. b) Simulation of interstitial flow generation from channels #1 to #3 over COL1 in channel #2. This simulation showed the velocity profile of flow resulting from a hydrostatic pressure of 60 µL between channels #1 and #3 during Step 3. c) Simulation of collagenase concentration applied to channel #3 and concentrated in channel #4 under the interstitial flow for 600 sec. After a loss of flow due to the equilibrium of a hydrostatic pressure (> 600 s), collagenase diffused from channels #4 to #3. However, collagenase did not reach channels #2 until 1800 s. d) Representative images of hydrogel channel selectively extracted from channel #4. Controls represented channels before cell isolation experiments. Cell‐embedded collagen scaffolds in the channel were observed in white under phase‐contrast microscope. In the case of success, since the cells and collagen gel were extracted, the white color of channel #4 became transparent; meanwhile, in the case of failure, channel #2, not an extraction target, was changed to completely (ii) or partially (iii) transparent.

During the 7 days of co‐culture, medium and cells in each channel were collected individually (**Figure** **2**a). Conditioned medium in channel #3 was first collected by pipette for cytokine profiling and hCMEC/D3 cells in channel #1 were trypsinized and collected. BM‐NSCLC cells embedded in COL1 in channel #4 and ACBRI 371 cells embedded in COL1 in channel #2 were then individually collected. To acquire only BM‐NSCLC cells in channel #4, collagenase was first injected to channel #3 with interstitial flow from channels #1 to #3 over the COL1 in channel #2. The flow protected the COL1 in channel #2 with ACBRI 371 cells from collagenase diffusion. Collagenase has a molecular weight range of 63–130 kDa and cannot diffuse against convective flow stronger than 3.95 × 10^−2^ mm s^−1^ (Figure 2b). Initial concentration of the applied collagenase was adjusted by the simulation to maintain the activating concentration of 0.5–2.5 mg mL^−1^ in channel #4 for more than 10 min (Figure 2c). COL1 only in channel #4 was successfully isolated to collagenase and melt (Figure 2d and Figure S3, Supporting Information). To prevent cell mixing, dissolved collagen in channel 2 was classified as a failure case. The developed protocol has a success rate of approximately 80%, 25 successes from 31 devices.

### 2.2 Increased Cytokine Secretion and Global Transcriptional Shift of Patient‐Derived Tumor Cells under Brain Microenvironment

**Figure 3:**
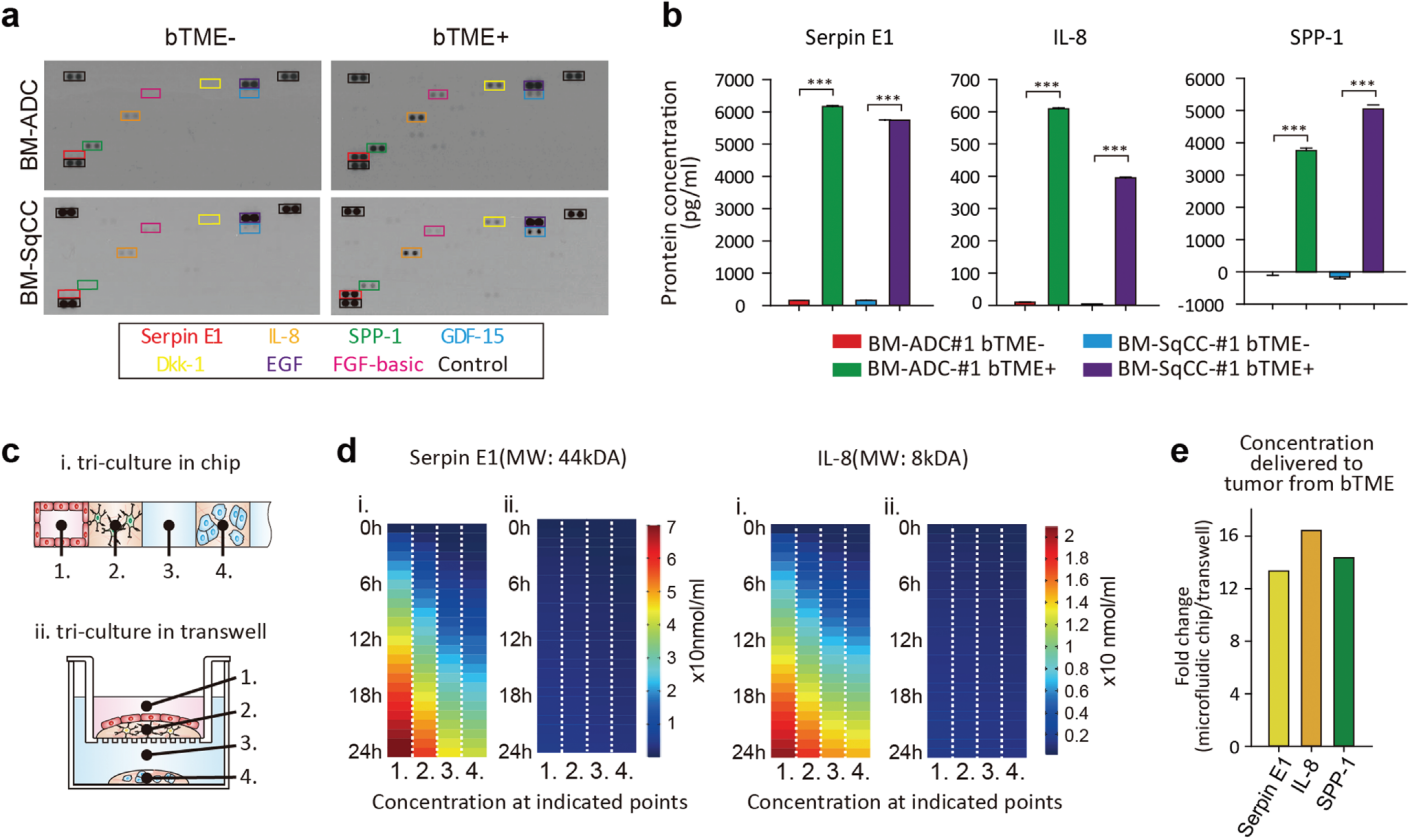
Cytokines elevated via microfluidic tri‐culture. a) Cytokine profile. Dots in the black boxes are references. b) Protein concentration measured in conditioned media extracted from microfluidic channel #3 with or without bTME. c) Schematic illustration for protein concentration simulation secreted from the cells in region 1, tri‐cultured in (i) a microfluidic device or (ii) Transwell. d) Simulated concentration of two proteins (large Serpin E1 and small IL‐8) in (i) a microfluidic device and (ii) Transwell. e) Ratio of concentration delivered to tumor cells in channel #4 in 24 h in a microfluidic chip to Transwell.

Cell culture supernatant was collected from the medium channel #3 between bTME components (channels #1 and #2) and BM‐NSCLC cells (channel #4) (Figure 1c); 24 h before collection, all growth media were fully refreshed with basal medium and incubated. Proteome profiler showed that three cytokines, serpin E1 (44 kDa), interleukin‐8 (IL‐8, 8 kDa), and secreted phosphoprotein 1 (SPP‐1, 32.9 kDa), were increased in bTME+ condition (Figure S4, Supporting Information), as confirmed via Luminex assay (**Figure** **3**b). Measured concentration values were normalized to those of the neurobasal medium (2.21 (serpin E1), 3.6 (IL‐8), and 5244.72 pg mL^−1^ (SPP‐1). The concentration levels of SPP‐1 in all bTME‐ cases were lower than the normalization control (cultured in neurobasal medium) due to the consumption by BM‐NSCLC cells. Molecules of 44 kDa mimicking the heaviest Serpin E1 reached BM‐NSCLC cells (channel #4) from bTME via diffusion by simulation (Figure 3c,d and Figure S4, Supporting Information). Small (8 kDa) IL‐8 secreted from bTME cells also diffused toward BM‐NSCLC cells in 12 h. The concentrations of both large and small cytokines in microfluidic device were tenfold larger than those in the Transwell (Figure 3e). Of the three cytokines, Serpin E1 and IL‐8 showed a strong correlation with reduced survival of lung ADC and SqCC patient groups in The Cancer Genome Atlas (TCGA) (Figure S5, Supporting Information).

**Figure 4:**
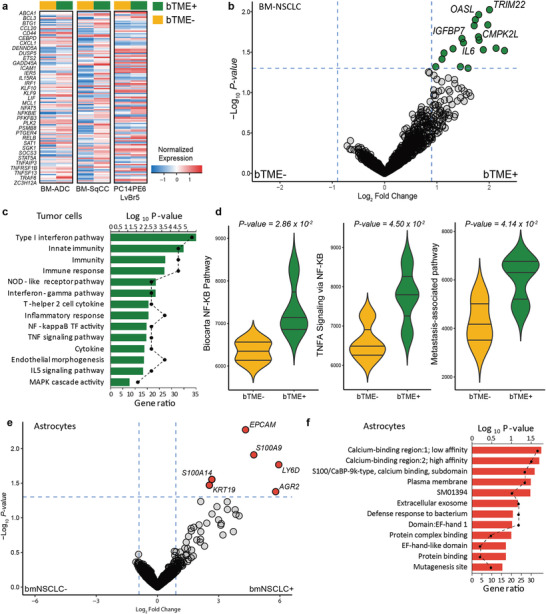
Transcriptome profiles of tumor cells and cells in TME. a) Identification of tumor cells’ mRNA expression bTME‐ versa bTME+. b) Volcano plot representation of a differential gene expression analysis showing the magnitude and significance of gene upregulations in tumor cells under bTME. c) GO analysis of hallmark genes enriched in BM‐NSCLC cells co‐cultured with bTME. d) Violin plots measuring the activity level of the NF‐κB related pathways and metastasis‐associated pathways using single sample gene set enrichment analysis (ssGSEA). e) Volcano plot representation of a differential expressed gene analysis showing the magnitude and significance of gene upregulations in astrocytes. f) GO analysis of hallmark genes up‐regulated in astrocytes co‐cultured under bTME.

Genome‐wide differential gene analysis was performed individually for BM‐NSCLC cells with or without bTME, hCMEC/D3 cells with or without BM‐NSCLC cells, and ACBRI 371 cells with or without BM‐NSCLC cells. Original genetic landscape of BM‐NSCLC cells used in this study is shown in Figure S6, Supporting Information. Target cells could be successfully collected in perfect selection from tri‐cultured population using the cell extraction protocol described above (Figure 2), and transferred for RNA sequencing. bTME+ BM‐NSNLC cells up‐regulated essential molecules associated with immune response, tumor necrosis factor (TNF) signaling pathway, and nuclear factor kappa‐light‐chain‐enhancer of activated B cells (NF‐κB) transcription factor activity (**Figure** **4**a–c). Activation of the NF‐κB signaling pathway was prevalent in our model (Figure 4a). Consistent with the previous observation, bTME+ BM‐NSCLC cells demonstrated enrichment of TNF*α* and metastasis‐associated pathways, further consolidating the previous notion that bTME facilitates BM^[^ ^21^ ^]^ (Figure 4d and Figure S6, Supporting Information).

The overall read counts for individual genes between BM‐NSCLC cells cultured with or without bTME demonstrated a strong correlation (Figure S7, Supporting Information). Global transcriptional profiles of ACBRI 371 cells in bTME revealed the enrichment of calcium‐binding activity^[^ ^22^ ^]^ and extracellular exosome^[^ ^23^ ^]^ with BM‐NSCLC cells (Figure 4e,f). Compared to BM‐NSCLC cells and astrocytes, hCMEC/d3 cells did not show apparent enrichment when cultured with BM‐NSCLC cells (Figure S8, Supporting Information).

### 2.3 Drug Response of Patient Tumor Cells under the Brain Tumor Microenvironment in Microfluidic Assay

**Figure 5:**
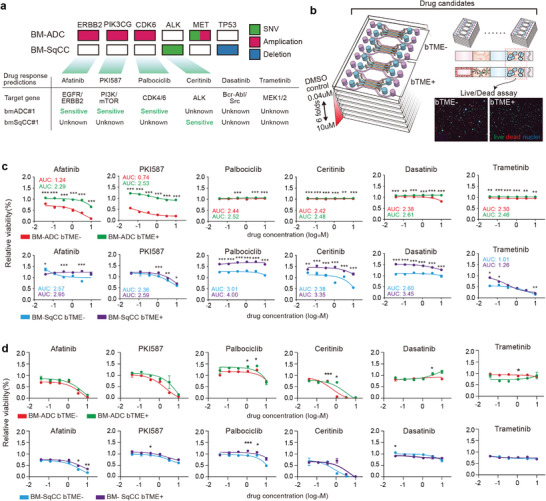
Drug screening in microfluidic chips. a) DNA mutation of BM‐NSCLC and DNA‐based drug response prediction. b) Schematic illustration of drug screening process using microfluidic chip. c) Drug response graphs calculated for tumor response with/without bTME using microfluidic chip. d) Drug response graphs using Transwell (*p < 0.05, **p < 0.01, and ***p < 0.001 coculture versus monoculture at each drug concentration).

Clinically actionable alterations responsive to ERBB2/ EGFR inhibitors, MAPK pathway inhibitors, CDK inhibitors, or inhibition of PI3K/Akt and the mTOR signaling, were shared in all BMs of patients. Six FDA‐approved and clinically available drugs were considered for the treatment of bTME+ and bTME‐ BM‐NSCLC cells, including afatinib (EGFR/ERBB2 inhibitor), PKI587 (PI3K/mTOR inhibitor), palbociclib (CDK4/6 inhibitor), Ceritinib (ALK inhibitor), dasatinib (Bcr‐Abl/Src inhibitor), and trametinib (MEK1/2 inhibitor). BM‐ADC cells had major mutations in ERBB2, PIK3CG, CDK6, MET, and BM‐SqCC showed a single number variation of ALK and deletion of TP53 (**Figure** **5**a and Figure S9, Supporting Information). Drug response predictions were preformed using cancerSCAN, a next generation sequencing based targeted deep sequencing analysis using a custom panel characterized with 5095 clinical samples.^[^ ^24^ ^]^ The cancerSCAN panel recommends two drugs with a sensitive response to afatinib and palbociclib in mutation of EGFR and CDK6 of BM‐ADC. Other mutations without clinical evidence of drug lists in cancerSCAN reports were marked as unknown.

The prediction was compared with the drug screening result of BM‐NSCLC cells with or without bTME in the microfluidic device (Figure 5b,c and Figure S10, Supporting Information) and a Transwell (Figure 5d). Treatment was applied for 7 days at a fixed concentration. bTME‐ BM‐ADC cells showed sensitive responses to Afatinib and PKI587, as predicted by DNA mutations, but resistant to palbociclib in contrast to the prediction. The firm resistance of bTME‐ BM‐ADC cells to ceritinib, dasatinib, and trametinib, was predicted using cancerSCAN. Palbociclib was resistant to bTME‐ BM‐ADC out‐of‐prediction. cancerSCAN estimated that genetic trait of BM‐SqCC to be wild and only ceritinib showed results. In our screening, bTME‐ BM‐SqCC presented dose‐dependent sensitivity to PKI587, ceritinib, and trametinib, which are possible drug candidates. Interestingly, bTME+ BM‐ADC cells showed acquired resistivity to afatinib and PKI587 (green color, Figure 5c). Effect of bTME enhancing viability (Figure 1e) was not clearly noticeable for the BM‐ADC cells. For BM‐SqCC cells, enhanced viability due to bTME was obvious. Meanwhile, bTME+ BM‐SqCC cells did not acquire resistivity to PKI587 or trametinib (purple color, Figure 5c). Responses varied from case to case and types of drugs and cells, with or without bTME. Biological mechanisms underlying complex cellular signaling pathways were not involved, in addition to the enhanced viability due to bTME. In a Transwell, BM‐NSCLC cells cultured also showed a good correlation between screening results and cancerSCAN predictions, although no significant changes in drug responses were observed when co‐cultured with bTME in Transwell (Figure 5d).

**Figure 6:**
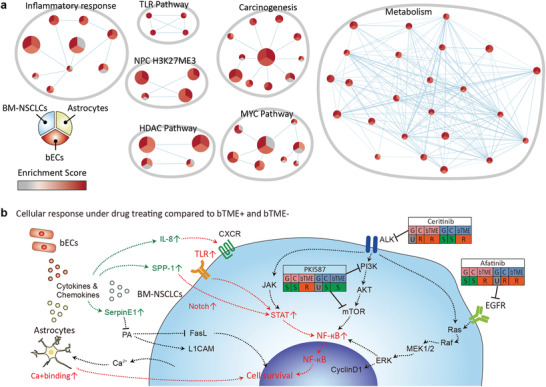
Summary of enriched genes and acquisition of drug resistance of cancer cells during bTME+ co‐culture. a) Cytoscape network map. Each node or circle represents a specific pathway that is enriched in either BM‐NSCLC, Astrocytes, or bECs. Red color indicates enrichment of the corresponding pathway and the node is divided into 3 separate segments, each segment reflecting activation of the pathway in BM‐NSCLCs, Astrocytes, or bECs. The size of the node reflects the number of genes that constitute each pathway, meaning that bigger nodes are comprised more genes compared to the smaller nodes. Last, two or more nodes are connected by an edge or a line if they share at least one gene in common. b) cellular responses to drug treatment and bTME. Black‐dashed lines indicate cellular pathways related to targeted drugs; red‐dashed lines indicate up‐regulated pathways induced by cytokines from bTME. The tables summarize drug screening results representing gene‐based prediction and cell‐based testing results in the microfluidic chip (G: gene‐based prediction, C: drug screening result without bTME, bTME: drug screening results with bTME. S: sensitive response, U: unknown, R: resistive response, red box: BM‐ADC, blue box: BM‐SqCC). Green arrows and characters mean increased cytokines and chemokines in bTME+. Red arrows and characters indicate increased genesets in bTME+.

Transcriptional regulatory networks that constitute cooperativity among tumor cells, endothelial cells, and astrocytes were identified using a global geneset enrichment analysis (**Figure** **6**a). Various essential cellular functions, including metabolism, inflammatory response, MYC, and histone deacetylase (HDAC) signaling pathways, were ubiquitously enriched in all three cell types, highlighting complex cellular dynamics that could potentially lead to transcriptional plasticity of BM‐NSCLC cells in the presence of bTME components. Possible procedures, selected drug responses, and cellular pathways related to the targeted drugs and cytokines are summarized (Figure 6b).

## 3 Discussions

The main aim of this study is to establish and analyze a landscape of the complicated interactions occurring at the metastatic site. The first constituent of the landscape is the enhanced viability of the bTME+ tumor cells, clearly observed only at the microfluidic scale. Secreted molecules from bTME are enriched near the tumor cells in microfluidic channels, thereby enhancing paracrine signaling.^[^ ^25^, ^26^ ^]^ Notch signaling pathway was activated in bTME+ tumor cells possibly by neighboring bECs,^[^ ^27^ ^]^ via a similar mechanism to the increased stem cell proliferation in perivascular niche (Figure S7, Supporting Information). Signaling loop of tumor cells and tumor‐associated astrocytes could also explain the enhanced viability. Notable enrichment of the calcium binding affinity marking tumor‐associated astrocytes might allow for the regulation of calcium ions in tumor cells and finally enabled protective effects (Figure 4f).^[^ ^22^ ^]^ However, the enhanced survival of bTME+ BM‐NSCLC cells cannot account for the selectively acquired viability enhancement only of bTME+ BM‐SqCC cells to palbociclib, ceritinib, and dasatinib.

Whole‐transcriptome analysis between tumor cells in the presence or absence of bTME demonstrated the second constituent of the landscape, distinct global gene expression profiles, where activation of NF‐κB pathway, immune and inflammatory response, and MAPK cascade activities were more prevalent in bTME+ co‐cultured tumor cells. Furthermore, tumor‐associated astrocytes were enriched with not only calcium‐binding affinity, but also extracellular exosomes (Figure 4f). These results are directly reflected upon cellular phenotypes where tumor cells harboring potential molecular targets such as genomic amplification of ERBB2 against afatinib and somatic mutation of PIK3CG against PKI587, and ALK mutations against ceritinib remained susceptible to respective target compounds. We noted an increase in the expression level of NF‐κB, a downstream of the ALK‐PI3K pathway, in co‐culture conditions, and hypothesized that paracrine cytokines signaling loops potentially promoted the alternative malignant transformation of tumor cells. Cytokine‐related NF‐κB activation seemed to increase the resistance of bTME+ BM‐SqCC to ceritinib and bTME+ BM‐NSCLC to afatinib. We also observed that a druggable candidate of PKI587 in both PDCs significantly acquired resistivity only on bTME+ BM‐ADC cells and not on BM‐SqCC cells which lack PIK3CG mutation, presuming that bTME activated an alternative signaling mechanism for PI3K. The NF‐κB activation might follow an alternative mechanism of PI3K inhibition, which should be verified in future studies. In the case of trametinib, we observed the death of hCMEC/D3 and ACRBI3. Trametinib caused broad stromal toxicity and hindered the observation of drug resistance changes by bTME.

The developed microfluidic model successfully mimicked the cytokine‐related intracellular pathways for the acquired drug resistivity by bTME.^[^ ^28^ ^]^ Metastatic NSCLC tumor cells express anti‐PA serpins (neuroserpin/serpinI1 and serpinB2) to prevent the intrinsic antitumor mechanism of astrocytes.^[^ ^6^, ^7^, ^29^ ^]^ The brain‐specific neuroserpin and the more broadly expressed serpins B2, E1, and E2 are selective inhibitors of plasmin.^[^ ^30^ ^]^ The most frequently upregulated serpins in brain‐seeking metastasis models were identified as neuroserpin and serpin B2, expressed in a high percentage of brain metastases from breast and lung cancer patients.^[^ ^30^ ^]^ In addition, Serpin E1 is involved in tumor migration via their complex signaling network with TGF‐*β*.^[^ ^31^ ^]^ IL‐8 activates the expression of Forkhead box C1 (FOXC1) via PI3K signaling to AKT and Hypoxia‐inducible factor 1‐alpha (HIF 1*α*).^[^ ^32^ ^]^ FOXC1 expression leads to transactivation of Interleukin 8 receptor, alpha (CXCR1), and chemokine (C‐C motif) ligand 2 (CCL2), thereby promoting inflammation and the invasive and metastatic abilities of inflammation‐associated cancer cells.^[^ ^32^ ^]^ Additionally, serpins can help tumor cells to avert Fas‐dependent programmed cell death and allow them to proliferate along brain capillaries, resulting in the engulfment and remodeling of a co‐opted capillary network.^[^ ^7^ ^]^ Global network analysis of BM‐NSCLC, hCMEC/D3, and ACBRI371 cells cultured in the microfluidic model showed increases in the inflammatory response, MYC, HDAC, carcinogenesis, and metabolism (Figure 6a) in correlation with previously reported MYC in brain metastatic lung ADC cells^[^ ^33^ ^]^ and HDAC in brain tumors.^[^ ^34^ ^]^ The results acquired with the developed microfluidic model further consolidated previous findings, including the inflammatory response in BM.^[^ ^28^ ^]^ The cytokine‐related NF‐κB activation seemed to cause an increase in MYC.^[^ ^35^ ^]^ The increased metabolism regulating efflux could release the drug from the cytoplasm.^[^ ^36^, ^37^ ^]^

The developed microfluidic model was proven to recapitulate the in vivo reciprocal communication of patient‐derived brain metastatic tumor cells with astrocytes and bECs on a miniature scale, with multiplexed high content imaging capability. The microscale dimension provided to the cell culture helped the secreted molecules from one cell to be enriched and efficiently transported to another.

In this study, we developed a unique cell and medium harvesting method from the microfluidic model and selectively collected each cell type after experiments for molecular expression profiling and deeper investigation. Advantages of the developed microfluidic model also exist in the spatial 3D reconstitution of ECM and multiple cell types and precise regulation of biochemical communication by robust and efficient transport. The microfluidic model provides a potential strategy for precise treatment and pre‐clinical evaluation of new therapeutic strategies against metastatic and resistive tumors. In particular, as immune signaling is activated, existing or newly developed immunotherapeutic approaches can be applied through co‐culture with PDCs and cancer‐associated immune cells.

## 4 Experimental Section

### Preparation of the Microfluidic Device

The fabrication protocols of the microfluidic device were previously reported.^[^ ^38^ ^]^ Briefly, SU‐8 photoresist 200 µm thick was first patterned on a silicon wafer using photolithography. Polydimethylsiloxane (PDMS, SYLGARD 184 Silicone Elastomer Kit, Dow Corning) was mixed with a curing agent to form PDMS pre‐polymer in a weight ratio of 1:10. The pre‐polymer was poured onto the patterned wafer and subsequently degassed in a vacuum chamber for 15 min to remove bubbles, and then baked in a drying oven at 80 °C for 4 h. The cured PDMS device was detached from the wafer, trimmed, and punched using a biopsy punch and blunt needle, cleaned with ethanol, and autoclaved at 120 °C for 30 min. The sterilized PDMS device was bonded with a cover glass by oxygen plasma treatment (FEMTO science) and dried in an oven at 80 °C for at least 24 h to recover hydrophobicity.

### Primary Cell Preparation

Immortalized human cerebral microvascular endothelial cell lines (hCMEC/D3, Ceullutions Biosystems Inc.) were maintained, subcultured, and preserved with Vasculife VEGF‐MV Endothelial Complete Kit (Vasculife‐MV, Lifeline cell technology). Normal human astrocytes (astrocytes, cell systems) were cultured in Complete Classic Medium with Serum and CultureBoost (Cell Systems) and Astrocytes Growth Medium BulletKit (AGM, Lonza), and preserved. To match the co‐culture medium of bTME, hCMEC/D3 and astrocytes were seeded in 6‐well plates at 2.0. × 10^5^ mL^−1^ in their culture media. In a day, various culture media were tested to determine the mRNA expression levels of the signature genes of endothelial and astrocytes using quantitative reverse transcription‐PCR. Patient tumor cells were isolated from donors using the methods previously described.^[^ ^18^ ^]^ The isolated tumor cells were cultured with Neurobasal Media (NBA, Gibco), which includes 2% B27 supplements (50X, Gibco), 1% N2 Supplement (100X, Gibco), 1% L‐glutamine (Gibco), 200 ng mL^−1^ of EGF (R&D systems), 200 ng mL^−1^ of bFGF (R&D systems), 30 ng mL^−1^ of rhNRG‐1‐b1/HRG‐b1(EGF domain) (R&D systems), and 200ng mL^−1^ of rhIGF‐1 (R&D systems) on non‐treated cell culture flasks (SPL) for suspension culture. The tumor cell spheres were grown to < 600 µm in diameter, cancer spheres were collected with culture media and washed with phosphate‐buffered saline (PBS, 1X, Gibco). The collected cell pellet was dissociated with Accutase (Gibco) to make a single cell and neutralized with PBS. Cells were proliferated and maintained in mouse xenografts, as previously described.^[^ ^18^ ^]^

### Multiple Cell Culture in the Microfluidic Model

Collagen solution was prepared by mixing the rat‐tail collagen type 1 solution (BD), 10×PBS, and distilled water (DW) to form the final concentration of the collagen gel at 2.0 mg mL^−1^. A very small amount of sodium hydroxide (NaOH, 0.1 N) was added to adjust the pH to 7.4. To prepare the cell‐collagen mixture solution, 20 µL of cells with medium of 1.0×10^7^ mL^−1^ (10 times higher than final cell density) and 180 µL of collagen solution of 2.22 mg mL^−1^ concentration were carefully mixed. Prepared collagen or collagen‐cell mixture solution was injected into the gel channel and incubated in a CO_2_ incubator at 37 °C for 30 min at high humidity. After gelation, basal or growth medium was added to the media channels. hCMEC/D3, which reached 80% confluency in the 75T flask, was collected with 0.05% trypsin and neutralized with Vasculife‐MV. Briefly, 60 µL of hCMEC/D3 suspension prepared at 8.0–10 ×10^5^ cells mL^−1^ in Vasculife basal media was added to the outer channel on both sides. After 2 h, the medium in the bEC culture channels was refreshed with 120 µL of fresh Vasculife‐MV for EC cell stabilization for less than 12 h. Communication channels were refreshed with 120 µL of fresh cancer growth medium. All medium channels were refreshed daily.

### Quantification of Cell Viability

A solution of Live/dead viability/cytotoxicity kit for mammalian cells (ThermoFisher Scientific) was mixed with basal media according to the manufacturer's protocol. The solution was introduced into the media channels. For homogeneous staining of cells in collagen gel, a live/dead solution‐filled microfluidic model was incubated in a 37 °C CO_2_ incubator with repeated tilting at 20° for 1 h. After the staining process, microchannels were washed twice with 1×FBS and incubated for 15 min to remove the remaining dye in the gel. Cell staining images were observed 4 to 5 in different regions of interest per array using a 4x objective lens. The number of stained cells was counted using ImageJ. And the viability was calculated by the following equation.

(1) $$\mathrm{viability}=\frac{\mathrm{the}\;\mathrm{number}\;\mathrm{of}\;\mathrm{live}\;\mathrm{cell}}{\left(\mathrm{the}\;\mathrm{number}\;\mathrm{of}\;\mathrm{live}\;\mathrm{cell}\;\mathrm{and}\;\mathrm{dead}\;\mathrm{cell}\right)}$$

The average of viability was from the number of regions of interest (BM‐ADC n = 65, BM‐SqCC n = 21)

### Cytokine Profiling with Medium in the Microfluidic Model

The cell culture supernatant from the microfluidic model was collected only from the communication channels; 24 h before collection, all growth media in the channels were completely refreshed with basal medium after washing twice. Cytokines in the co‐culture supernatant were detected using a Proteome Profiler Human XL Cytokine Array Kit (R&D Systems) according to the manufacturer's protocol. The mean pixel density of blotting was measured using ImageJ (NIH). To measure the concentration of the selected proteins, bead‐based multianalyte profiling using cell culture supernatants was performed using the Luminex assay (R&D systems) for the selected three panels, serpin E1, IL‐8, and SPP‐1. The cell culture supernatants were collected from 3 chips. And each supernatant was diluted twofold and analyzed.

### Simulation of Convection and Diffusion Model in Fluidic Platform

The simulations were computed using the microfluidics module of Comsol 5.6, and calculated diffusion coefficients of each molecules using Equation (2) described by Erickson,^[^ ^39^ ^]^ Stokes–Einstein equation (Equation (3)),^[^ ^40^, ^41^ ^]^ and Arrhenius relationship (Equation (4)) with properties of collagen described here.^[^ ^42^, ^43^ ^]^

(2) $$r=0.66M^{\frac{1}{3}}$$

(3) $$D=\frac{k_{b}}{6\pi r\mu }$$

(4) $$\frac{D_{\mathrm{collagen}}}{D_{\mathrm{water}}}=\exp\left(-\emptyset^{\frac{1}{2}}\frac{r}{r_{f}}\right)$$

M is the molecular weight of protein, r is the effective radius of proteins, D is diffusion coefficient of protein, *k*_b_ is the Boltzmann's constant, *T* is kelvin temperature, μ is the dynamic viscosity of the water, *D*_collagen_ is diffusion coefficient of protein in collagen, *D*_water_ is diffusion coefficient of protein in water, ø is the volume fraction (0.125), and *r*_f_ is the radius of type 1 collagen (200nm).^[^ ^44^ ^]^ Therefore, the diffusion coefficients of collagenase were assumed to be 8.4 × 10^−11^ m^2^ in medium channels (Channels #1, #3, and #5) and 8.35 × 10^−11^ m^2^ s^−1^ in collagen channels (Channels #2 and #4). The initial concentration of collagenase was 7 × 10^−3^ mol m^−3^ (0.5 mg mL^−1^) in channels #3. The velocity was generated by a hydrostatic pressure of 60 µL, between reservoir channels #1 and #3. Due to the flow of channels #1 to #3 through the collagen, the initial hydrostatic pressure reached equilibrium after 10 min.

To simulate the cytokine secretion and diffusion model, the cytokine secretion rate was first calculated in cells per hour based on the values measured in the Luminex assay.

Based on the values calculated in **Table** **1**, a simulation model was designed with cells in the chip and Transwell. In the simulation, the number of cells placed in the chip and Transwell was the same as the number of cells seeded in the experiment. The diffusion coefficient of cytokines was calculated based on Equations (2)–(4). The simulation model generated a model of cytokine secretion and diffusion over 1 day.

**Table 1:** Estimates of cytokines secreted per day by cells calculated based on luminex measurements

	Serpin E1 [µmol cell^−1^ day^−1^]	IL‐8 [nmol cell^−1^ day^−1^]
BM‐ADC	0.673	221
BM‐SqCC	0.654	78.96
hCMEC/D3	1.92	64.0
ACBRI 371	0.975	93.12

### Selective Cell Isolation from the Microfluidic Model

To isolate tumor cells embedded in the collagen gel in the center ECM channel, collagenase solution with collagenase type D (Roche) prepared to 1.0 mg mL^−1^ in PBS was utilized; 60 µL of pre‐warmed collagenase solution was only filled in the communication channels, and 120 µL of cold PBS was filled into the outer media channels to generate pressure‐driven continuous flow from media channels to communication channels, to selectively degrade ECM with tumor cells, not one with astrocytes. Cold PBS was important to protect the ECM from astrocytes. After incubation in a CO_2_ incubator at 37 °C for 20–30 min (Figure S6b–d, Supporting Information), dissolved collagen gels, and tumor cells in the central channel were collected using a pipette in a conical tube. The collection was centrifuged at 1000 rpm for 5 min to remove the supernatant and isolate pellets. From the washed cell pellet, DNA was isolated using Trizol solution (Thermo Fisher Scientific) and RNA using Trizol and RNeasy mini kit according to the manufacturer's protocol. The concentration and purity of both DNA and RNA were measured using a Nanodrop 2000 UV–vis spectrometer (Thermo Scientific).

### Whole Transcriptome Sequencing

TruSeq RNA Sample Prep Kit (Illumina) was used to prepare RNA‐Seq libraries and HiSeq4000 (Illumina) was used to generate paired‐end 100 bp reads. The sequenced reads from DNA and RNA sequencing were mapped to hg19 using the Burrows–Wheeler Aligner (BWA) and GSNAP.^[^ ^45^, ^46^ ^]^ Expression levels for RNA‐seq data were estimated with DEGseq by reading per kilobase million (RPKM) values of 23 660 human Ref Seq genes.^[^ ^47^ ^]^ The obtained RPKM values were log2 transformed, and log2 (RPKM+1) values were used in further analyses. Single‐sample GSEA (ssGSEA) scores were calculated using the GSVA R package for oncogenic pathways from the mSigDB geneset database.^[^ ^48^, ^49^ ^]^

### Analysis of Differentially Expressed Genes

DESeq2 extracted 114 significantly up‐regulated genes in cells from the multi‐culture condition compared to those from the monoculture condition (adjusted p‐value < 0.05).^[^ ^50^ ^]^ DAVID provided gene ontology (GO) terms that were associated with the extracted genes,^[^ ^51^, ^52^ ^]^ and the GO network was drawn using the enrichment map in Cytoscape.^[^ ^53^, ^54^ ^]^ Transcription factors of these 114 genes were estimated by iRegulon in Cytoscape.^[^ ^54^, ^55^ ^]^ Unsupervised clustering was performed using the Complex Heatmap (R package) with Euclidean distance and complete linkage clustering methods.

### Drug Efficacy Estimation and Screening Using the Microfluidic Model

Six types of drugs for BM cases in clinics with various target genes were selected. Every inhibitory drug was purchased from Selleck Chemicals (Houston, TX, US). Each stock was prepared with 1 mm concentration in dimethyl sulfoxide (DMSO, Sigma‐Aldrich) and mixed in culture media with 6 segments at concentrations of 0.04–10 µm. Each volume of DMSO was the same at various concentrations of the drug. Fresh drug solutions were supplied to the media reservoir and replaced daily for 7 days. Drug screening was set up on 2–3 chip arrays for each condition as shown in Figure 5b. Viability was measured and calculated using the same method as in method 4.4. The viability of the entire experiment for each drug condition was normalized to DMSO control at the endpoint of drug. The viability of the entire experiment for each drug condition was normalized to 1mm DMSO in culture media condition at the endpoint of drug screening in each group. The viability ware averaged and presented in drug response graph (n ≥ 8). For Transwell drug screening, drug evaluation was performed in 24 wells plate to use the same amount of culture medium as microfluidic chip. Each condition was performed in duplicate. Drug evaluation was conducted for 7 days, and after the end of the experiment, viability was measured in the same way as method 4.4.

### Collection of Patient‐Derived Specimens and Ethics Approval

Fresh tumor specimens were obtained from brain metastatic NSCLC patients via surgical resection at the Samsung Medical Center (Seoul, Republic of Korea) in accordance with the guidelines of the institutional review board (#201 004 004). Informed consent for each patient was obtained prior to the study.

## Conflict of Interest

The authors declare no conflict of interest.

## Author Contributions

H.K. and J.K.S. contributed equally to this work. H.K. designed the research, developed and performed the experiments, analyzed the data with assistance from J.K., H.J.O., and D.E.J., and wrote the manuscript. J.K.S. and H.J.C. analyzed genomic and transcriptomic data and wrote the manuscript. D.‐H.N. and H.W.L. provided concept of the study. D.‐H.N. provided biospecimens and clinical interpretation. H.W.L. and S.C. designed the research, analyzed the data, and wrote the manuscript.

## Supporting information

Supporting Information

## Data Availability

The data that support the findings of this study are available from the corresponding author upon reasonable request.
