# Supplementary material for: Recapitulated Crosstalk between Cerebral Metastatic Lung Cancer Cells and Brain Perivascular Tumor Microenvironment in a Microfluidic Co‐Culture Chip
Source: Adv Sci (Weinh). 2022 Jun 3;9(22):2201785. doi: 10.1002/advs.202201785 (PMC9353479; doi:10.1002/advs.202201785)
Supplement: Supplementary file 1 — Supporting Information [file ADVS-9-2201785-s001.pdf]

ADVANCED SCIENCE
Open Access

Supporting Information

for *Adv. Sci.*, DOI 10.1002/advs.202201785

Recapitulated Crosstalk between Cerebral Metastatic Lung Cancer Cells and Brain Perivascular Tumor Microenvironment in a Microfluidic Co-Culture Chip

Hyunho Kim, Jason K. Sa, Jaehoon Kim, Hee Jin Cho, Hyun Jeong Oh, Dong-Hee Choi, Seok-Hyeon Kang, Da Eun Jeong, Do-Hyun Nam, Hakho Lee, Hye Won Lee and Seok Chung**

Supporting Information

Recapitulated crosstalk between cerebral metastatic lung cancer cells and brain perivascular tumor microenvironment in a microfluidic co-culture chip

Hyunho Kim†*, Jason K. Sa*†*, Jaehoon Kim, Hee Jin Cho, Hyun Jeong Oh, Dong-Hee Choi, Seok-Hyeon Kang, Da Eun Jeong, Do-Hyun Nam, Hakho Lee, Hye Won Lee*, Seok Chung**

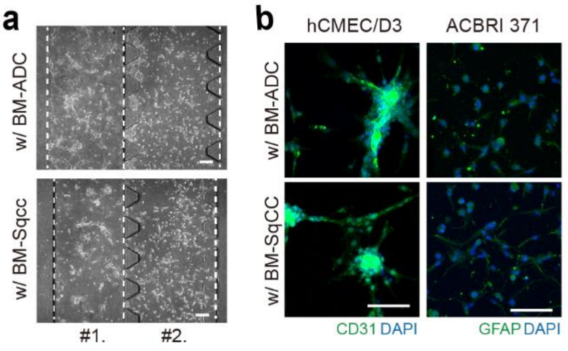

Fig. S1. Morphological analysis of cultured cells in a microfluidic device. a, Merged phase-contrast images of brain-specific stromal cells cultured 7 days in microfluidic channels with cancer cell culture medium (#1; hCMEMC/D3, #2; ACBRI 371). **b,** Representative fluorescence images of hCMEC/D3 and ACRBI 371 stained individually with CD31 and GFAP (both green colors).

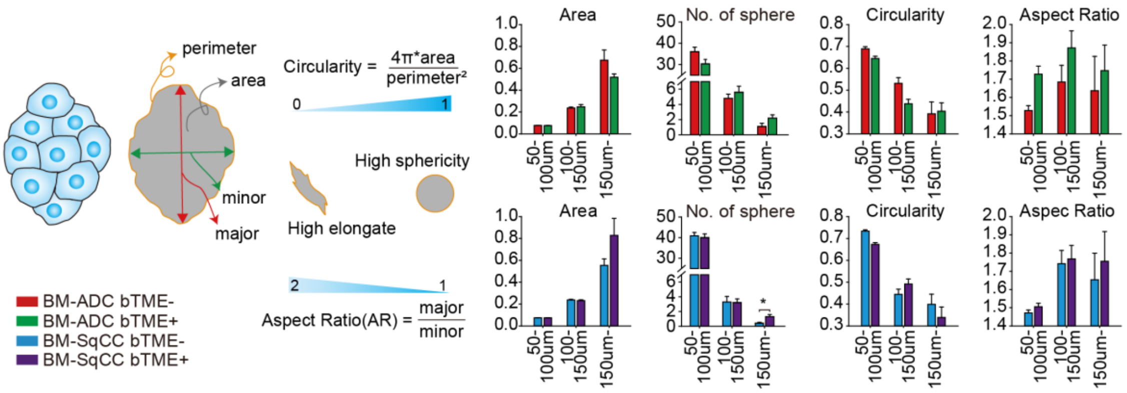

Fig. S2. Morphological indices of tumor cell spheres. Four indices were measured to monitor the phenotypic changes in the morphology of aggregated BM-NSCLC cells.

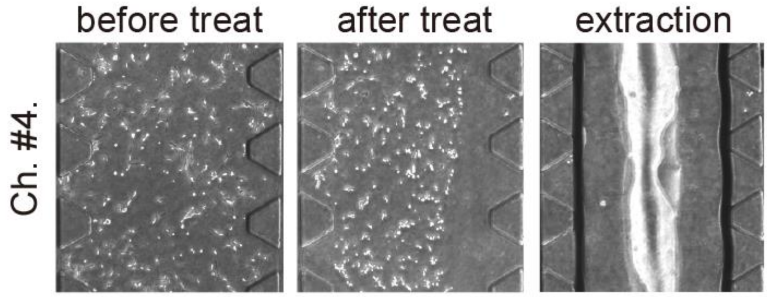

Fig S3. Phase-contrast images for BM-NSCLC containing hydrogel collection. (left) Type 1 collagen hydrogel with BM-NSCLC cells before collagenase treatment in channel #4. (center) Hydrogel collapse in 30 min of collagenase treatment. (right) Hydrogel is completely melted and ready for extraction.

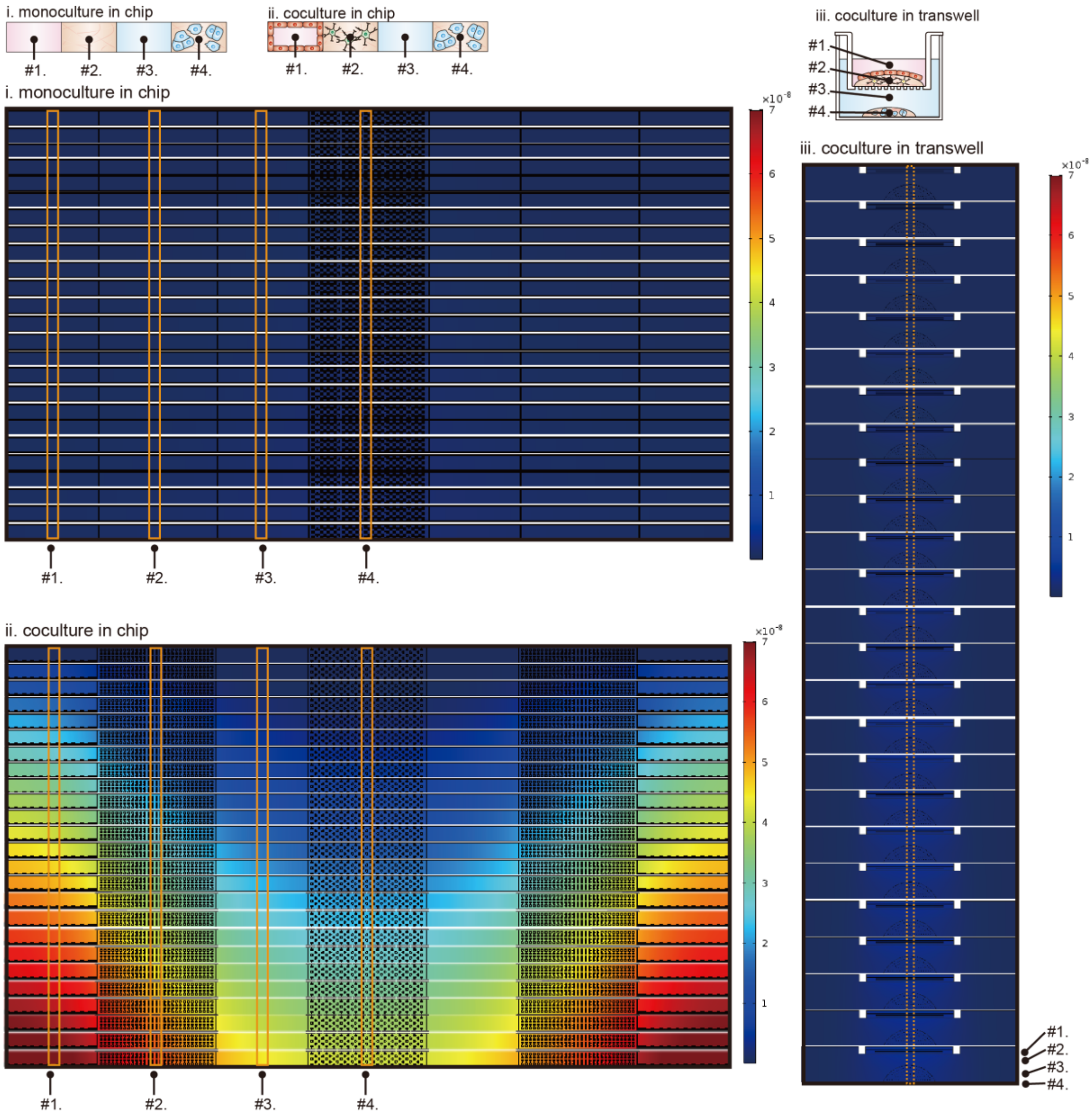

Fig. S4. Simulated concentration of secreted cytokines from the cells in channels #1 and #2 in a microfluidic chip and in regions #1 and #2 in Transwell assay. Orange boxes indicate the center position of each microchannel.

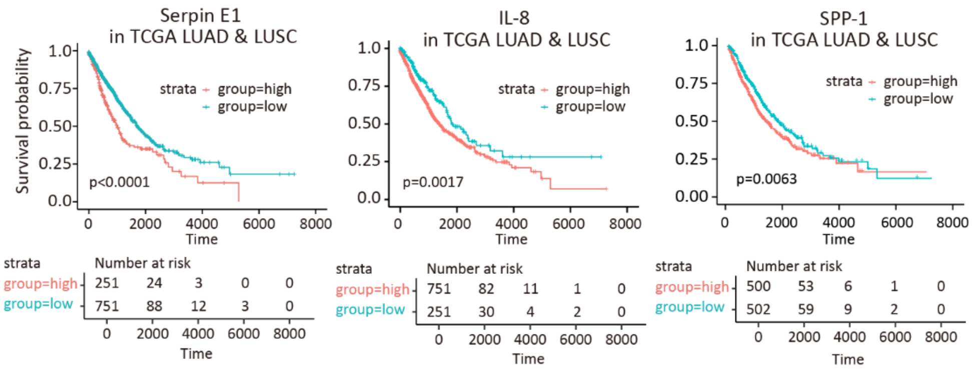

Fig. S5. TCGA survival plots for the patients groups expressing Serpin E1, IL-8, and SPP-1.

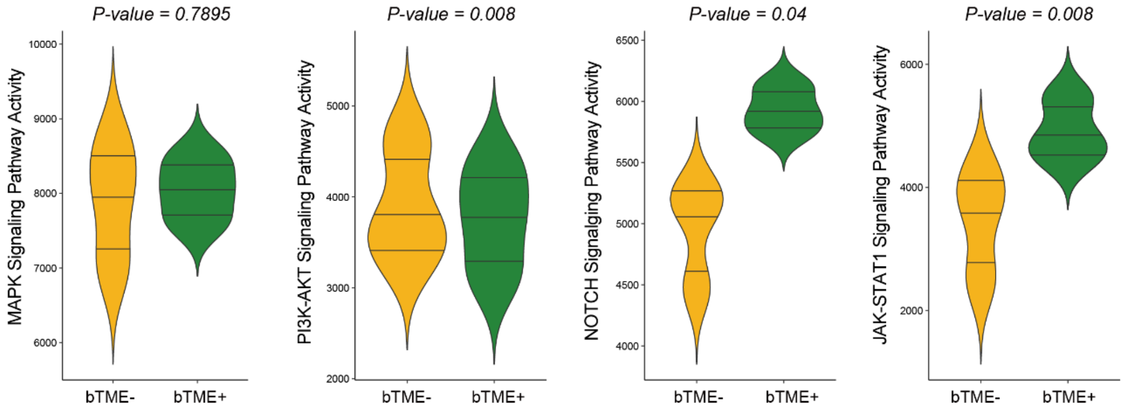

Fig. S6. Violin plots indicating the activity level of the MAPK, PI3K-AKT, NOTCH, and JAK-STAT1 pathways of BM-NSCLC using single sample gene set enrichment analysis (ssGSEA).

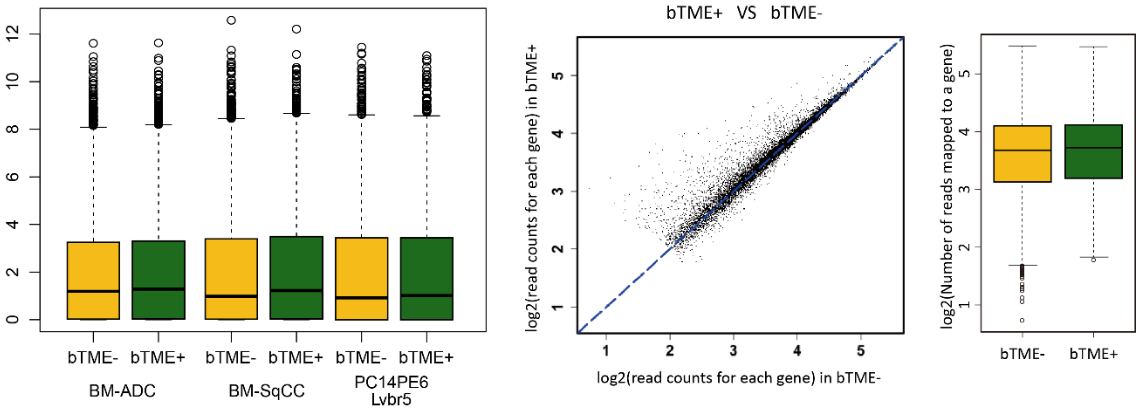

Fig. S7. Number of total reads from RNA sequencing of each sample. (left) Total reads without (orange) or with (green) bTME (center and right). The overall read counts for individual genes between BM-NSCLC cells co-cultured with bTME and without bTME.

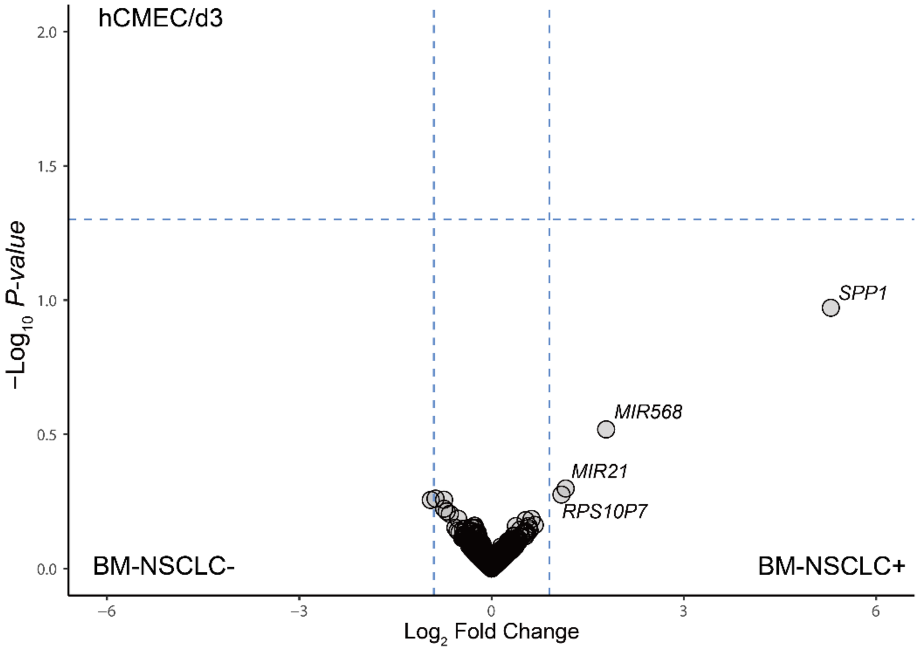

Fig. S8. Volcano plot of hCMEC/D3 cells. Differential gene expression analysis showing magnitude and significance of gene upregulations in hCMEC/d3 with or without BM-NSCLC cells.

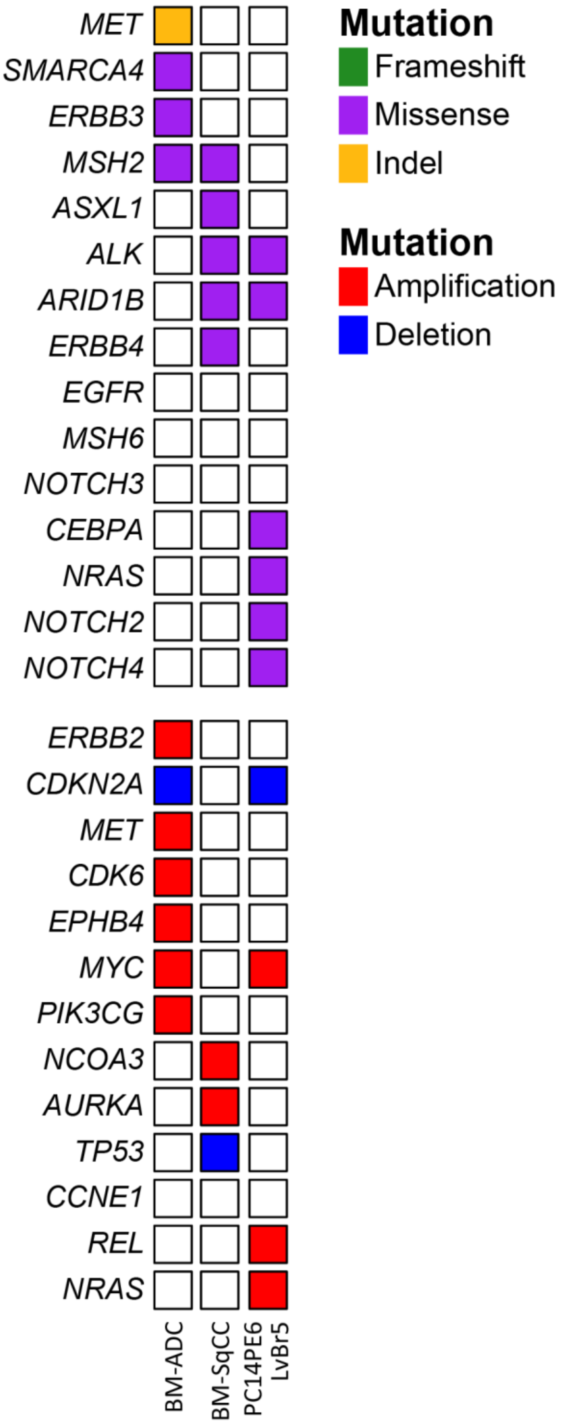

Fig. S9. Genetic landscape of BM-NSCLC cells used in this study.

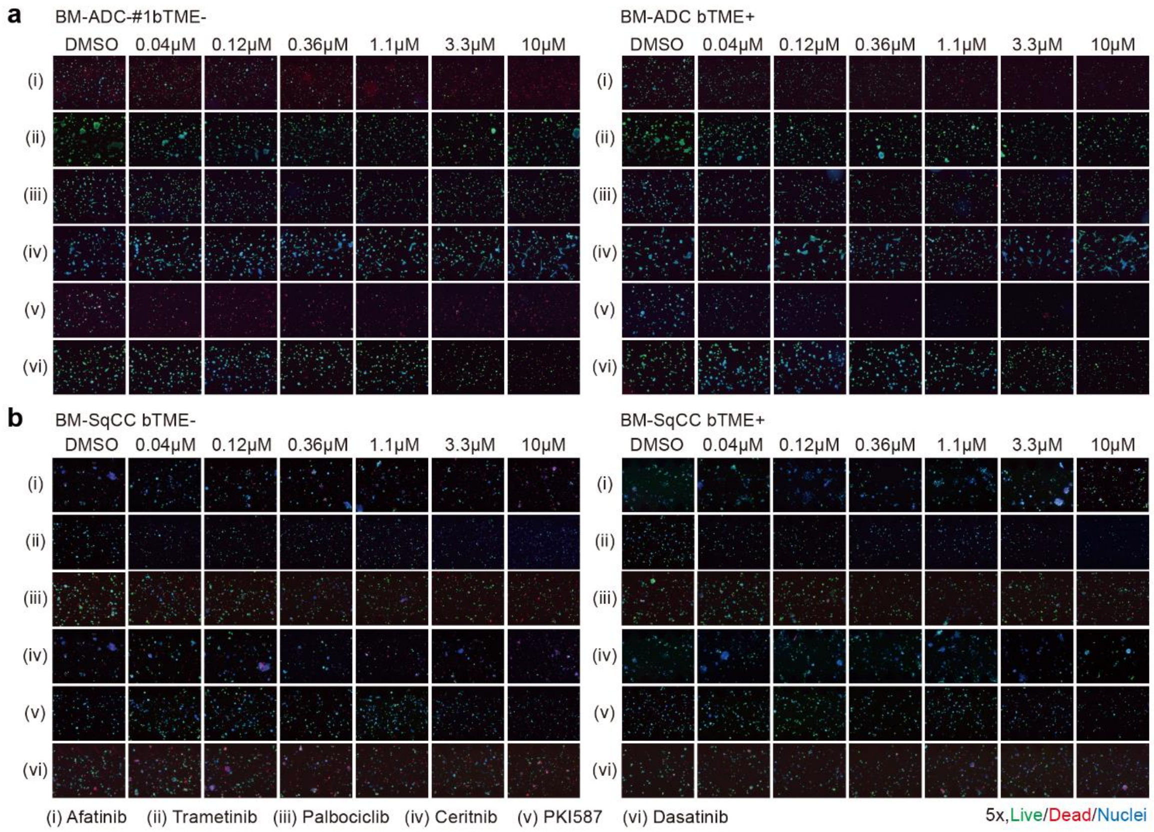

Fig. S10. Original drug screening images of BM-NSCLC cells. Live-dead fluorescent images for six drugs on BM-NSCLC cells in microfluidic device with or without bTME.
